# Amoxicillin vs. placebo to reduce symptoms in children with group A streptococcal pharyngitis: a randomized, multicenter, double-blind, non-inferiority trial

**DOI:** 10.1007/s00431-024-05705-1

**Published:** 2024-08-31

**Authors:** Renato Gualtieri, Charlotte Verolet, Chiara Mardegan, Sébastien Papis, Natasha Loevy, Sandra Asner, Marie Rohr, Juan Llor, Ulrich Heininger, Laurence Lacroix, Laure F. Pittet, Klara M. Posfay-Barbe

**Affiliations:** 1 Division of General Pediatrics, Department of Pediatrics, Gynaecology and Obstetrics, Geneva University Children’s Hospital and University of Geneva, Geneva, Switzerland; 2 Pediatric Infectious Diseases and Vaccinology Unit, Lausanne University Hospital, Lausanne, Switzerland; 3 Department of Pediatrics, Sion Hospital, Centre Hospitalier du Valais Romand, Sion, Switzerland; 4 Department of Pediatric Infectious Diseases and Vaccinology, University Children’s Hospital Basel, Basel, Switzerland; 5 Department of Pediatric Emergency Medicine, Geneva University Children’s Hospital and University of Geneva, Geneva, Switzerland; 6 Pediatric Infectious Diseases Unit, Geneva University Children’s Hospital and University of Geneva, Geneva, Switzerland

**Keywords:** Group A Streptococcus, Amoxicillin, Placebo, Streptococcal pharyngitis

## Abstract

The efficacy of antibiotic therapy for group A streptococcus (GAS) pharyngitis is debated. The role of antibiotics in preventing complications seems limited, with the main potential benefit being symptom duration reduction. Our study aimed to evaluate whether a placebo is non-inferior to amoxicillin in reducing fever duration. We randomized 88 children between 3 and 15 years of age presenting with acute symptoms of pharyngitis and a positive rapid antigen detection test for GAS to receive 6-day treatment with either placebo (*n* = 46) or amoxicillin (*n* = 42). The primary outcome was the difference in fever duration, with a non-inferiority threshold set at 12 h. The secondary outcomes included pain intensity and complications of streptococcal pharyngitis. The mean difference in fever duration between the amoxicillin and placebo groups was 2.0 h (95% CI, − 8.3 to 12.3) in the per-protocol analysis and 2.8 h (95% CI, − 6.5 to 12.2) in the intention-to-treat analysis. Treatment failure was observed in six participants in the placebo group and two in the amoxicillin group (relative risk, 2.15; 95% CI, 0.44–10.57). All patients were identified early and recovered well. There was no clinically relevant difference in pain intensity between groups over the 7 days following randomization, with the largest difference of 0.5 (95% CI, − 0.62–1.80) observed on day 3. *Conclusion*: Placebo appears to be non-inferior to amoxicillin in reducing fever duration. Pain intensity and risk of complications were similar between the two groups. These findings support the restrictive antibiotic treatment for streptococcal pharyngitis.

**Table:** 

**What is Known:** • *Group A streptococcus pharyngitis is a common reason for prescribing antibiotics in pediatric care.* • *In high-income countries, while antibiotic treatment has not been effective in preventing non-suppurative complications, the primary justification for their use remains the reduction of symptoms.*
**What is New:** • *Our results suggest that antibiotics have a limited impact on the duration of fever and the intensity of pain in children with streptococcal pharyngitis.* • *Considering that suppurative complications can be promptly treated if they arise, we recommend a more judicious approach to antibiotic prescriptions.*

*Trial registration*: The trial is registered at the US National Institutes of Health (ClinicalTrials.gov) # NCT03264911 on 15.08.2017.

**Supplementary Information:**

The online version contains supplementary material available at 10.1007/s00431-024-05705-1.

## Introduction

Pharyngitis is a leading cause of primary care outpatient visits among adults and children. Most cases are caused by viruses, such as rhinovirus, coronavirus, and adenovirus [1]. Group A streptococcus (GAS) is the most common bacterial cause of acute pharyngitis, [2] accounting for 5–15% of cases in adults and 20–30% in children. It most often affects children 5 to 15 years of age during the winter months and is rare in children under 3 years old [3]. Symptoms of GAS pharyngitis typically resolve spontaneously within 3–5 days [4]. However, it can be followed by suppurative complications such as acute otitis media, acute sinusitis, cellulitis, and quinsy, as well as non-suppurative complications such as acute rheumatic fever and post-streptococcal acute glomerulonephritis.

Based on the literature from the 1960s and 1970s, [5, 6] antibiotic therapy was routinely recommended in 2012 by the Infectious Diseases Society of America for all confirmed cases of GAS pharyngitis to reduce the risk of suppurative and non-suppurative complications and the duration of symptoms [7]. These guidelines are currently under review, and the effectiveness of antibiotic therapy in treating and preventing complications of streptococcal pharyngitis has been widely questioned in the literature over the past decade [8]. Since the beginning of the twenty-first century, several European countries (including Belgium, the United Kingdom, and the Netherlands) have changed their recommendations and now advise symptomatic treatment only, limiting the use of antibiotics to well-defined cases [9–12].

While antibiotic treatment remains unquestionable in low-income countries where the incidence of rheumatic fever is high and the sequelae from GAS infections have a significant impact on public health, [13] in high-income countries, reducing the intensity and duration of the symptoms of GAS pharyngitis may currently be the only reasonable justification for antibiotic therapy. A recent systematic review on the management of pharyngitis, mainly including studies involving adults, showed modest effectiveness of antibiotics on symptom resolution and concluded that there is insufficient evidence to draw conclusions in children [14]. In Switzerland, at the initiation of our study, suspected cases of streptococcal pharyngitis in pediatric patients over 3 years old with a McIsaac score exceeding 3 prompted a rapid test. Positive results led to the systematic initiation of amoxicillin treatment at 50 mg/kg/day for 7 days. However, the Swiss Society for Infectious Diseases has since released updated national recommendations, [15] advocating primarily for symptomatic treatment. Antibiotic treatment is now advised only in very specific and limited cases, where there is a risk factor of complications such as poor general condition, suspicion of an abscess, immunosuppression, personal or family history of rheumatic fever, recent immigration from a low-income country, or cardiac valve disease [16].

Our study focuses on a population of healthy children aged between 3 and 15 years with GAS pharyngitis. The primary objective of this study was to assess whether placebo treatment is non-inferior to amoxicillin in reducing the duration of fever in children with GAS pharyngitis.

## Methods

From January 2017 to May 2021, we conducted a prospective, double-blind, randomized, non-inferiority clinical trial in children with GAS pharyngitis who presented to the emergency departments of two pediatric university hospitals and one regional hospital in Switzerland.

The study was approved by all local ethics committees (CCER 15–086) and followed the International Council for Harmonization Good Clinical Practice guidelines and the Declaration of Helsinki. Written informed consent was obtained from parents or legal guardians after a detailed explanation of the study was provided by trained study staff. The study was overseen by an independent data safety and monitoring board. We followed the Consolidated Standards of Reporting Trials (CONSORT) reporting guidelines.

### Study population

Inclusion criteria were healthy children aged between 3 and 15 years with fever > 8 °C, clinical symptoms suggestive of pharyngitis, a McIsaac score ≥ 3, [17–19] and a rapid antigen detection test positive for GAS. Exclusion criteria were beta-lactam hypersensitivity/allergy; another concomitant disease that needed to be treated with antibiotics; immunological deficiency; oncological disease; chronic heart, liver, or kidney disease; antimicrobial therapy started within 72 h prior to study enrolment; a previous history or family history of acute rheumatic fever; skin infection suggestive of GAS impetigo; suspected post-streptococcal acute glomerulonephritis; complicated pharyngitis (e.g., pharyngeal abscess); or a rash suggestive of scarlet fever.

### Randomization and blinding

Children were randomly assigned in a 1:1 ratio to receive either a 6-day placebo regimen (intervention group) or amoxicillin tablets (control group). The amoxicillin dose was adjusted according to the participant’s weight to achieve the recommended dose of 50 mg/kg/day in two doses [20, 21]. For children weighing < 18 kg, the dose was 375 mg twice daily; for those weighing between 18 and 24 kg, the dose was 500 mg twice daily; and for those weighing > 24 kg, the dose was 750 mg twice daily. Tablets were identical in appearance, odor, taste, and packaging. Randomization was stratified by weight groups (< 18 kg, 18–24 kg, and > 24 kg) and study centers using block sizes of 2, 4, and 6. The randomization allocation sequence was generated by the Clinical Trials Pharmacy Unit of Geneva University Hospitals using dedicated software, which also labeled the study medication. Apart from the unblinded pharmacists and trial monitors, all members of the study team were blinded to the treatment group assigned to each child. Participants in both groups also received a prescription for paracetamol and nonsteroidal anti-inflammatory drugs (NSAIDs) for the symptomatic treatment of fever and pain.

### Study procedure

After inclusion, demographic and clinical data were collected, and a standardized physical examination and throat culture were performed. Following these procedures, treatment according to the allocation group was distributed to parents, who were instructed to start immediately. During the 7 days after randomization, parents were instructed to measure the fever twice daily using an axillary thermometer, closely monitor the evolution of symptoms (once daily), and record them in a diary, as well as every administered dose of paracetamol or NSAIDs. Symptoms collected included sore throat, abdominal pain, diarrhea, nausea, vomiting, anorexia, asthenia, and skin rash. Caregivers received a telephone call on day 3 after randomization for a standardized assessment. A control visit, including a repeated throat culture and clinical evaluation, was scheduled 1 month after randomization, as well as completion of a standardized questionnaire. Two phone calls were scheduled at the 6- and 12-month follow-ups to evaluate relapse, recurrence, and/or potential late complications of GAS pharyngitis. The throat culture performed immediately after inclusion aimed to identify the subgroup of patients with culture-confirmed GAS pharyngitis, given the risk of false-positive RADT results. The second throat culture performed at the one-month follow-up visit enabled the measure of GAS eradication, one of the secondary endpoints of the study. The research team, clinicians in charge, and the patients were all blinded to the culture results.

### Outcomes

The primary outcome was the difference in the fever duration. Secondary outcomes included pain intensity, use of symptomatic treatment, treatment failure, persistence of symptoms on day 3, GAS pharyngitis complications, and GAS eradication rate assessed 1 month after randomization. Pain intensity was assessed using the Faces Pain Scale-Revised (FPS-R) [22]. Treatment failure was defined as the occurrence of any complication associated with GAS pharyngitis or any change or deterioration upon clinical examination before the end of the 6-day treatment that warranted trial discontinuation and prescription of antibiotic therapy. The emergence of a rash suggestive of scarlet fever was therefore considered an indication to discontinue the study and administer antibiotic therapy.

Adverse events were assessed, recorded, and treated until resolution or stabilization. They were reported for review and acted upon within 24 h.

### Power calculation and statistical analysis

We estimated that 99 patients per treatment arm ensured that the study had 90% power to determine the non-inferiority of placebo on the duration of fever (with a non-inferiority margin of 12 h), based on the assumption that the standard deviation of fever duration is 1.2 days [23].

Descriptive statistics were used for patient demographics and baseline data, which were summarized by frequencies and proportions for categorical variables and means and SDs for continuous variables.

Primary analysis was performed in both per-protocol (PP) and intention-to-treat (ITT) populations. While ITT analysis is favored for superiority trials, PP is often recommended for non-inferiority trials because of the potential for an ITT analysis to increase the type I error [24, 25] and the Food and Drug Administration and European Medicines Agency recommend reporting both the PP and ITT in non-inferiority trials [26, 27]. Concordance between both analyses lends enhanced credibility and robustness to the findings [24, 28]. Mann–Whitney *U* test was used to assess non-inferiority in responses between the two groups with 95% CIs around the difference; if the upper limit of the 95% CI for this difference was less than 12 h, the placebo could be considered as non-inferior to amoxicillin. A sensitivity analysis was conducted in the subgroup of participants with culture-confirmed GAS pharyngitis.

Fever duration was also evaluated in a survival analysis, in which participants with incomplete follow-up were censored when they discontinued the intervention or withdrew. The mean difference and 95%CI of pain scores of each group were calculated by the group for each of the 7 consecutive days following randomization. The use of symptomatic treatment in the first 3 days following randomization, presence of clinical symptoms at day 3, treatment failures, complications during the 12-month follow-up, and negative throat culture 1 month after randomization were compared between groups using the chi-square test and Fisher’s exact test, as appropriate. Logistic regression was used to identify associated factors when any statistically significant difference was found. Statistical analyses were conducted using Stata v17.0 (StataCorp. software (College Station, TX, USA).

## Results

**Fig. 1:**
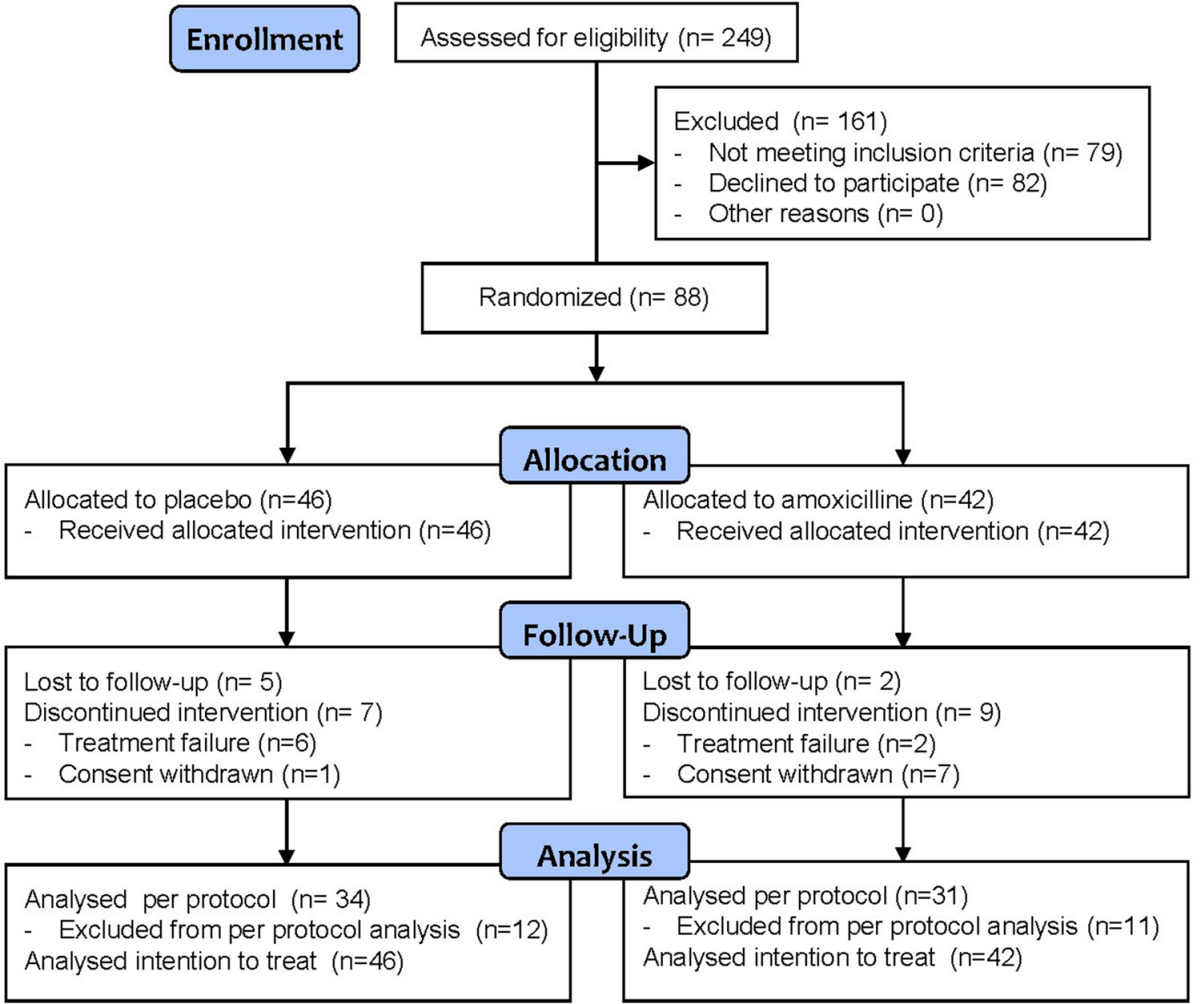
CONSORT diagram

Of the 249 children screened, 79 (32%) were ineligible and 82 (33%) refused to participate. A significant reluctance by parents to accept the randomized, double-blind design, in addition to the ongoing COVID-19 pandemic, negatively impacted recruitment, and the trial ended prematurely in May 2021, without reaching the target sample size. In total, 88 children were randomly assigned to the treatment groups: 42 to amoxicillin and 46 to placebo. Baseline demographic and clinical characteristics were comparable between the two groups (Table 1). A total of 34 children in the placebo group (74%) and 31 children in the amoxicillin group (74%) adhered to the scheduled 6 days of therapy and were included in the PP analysis (Fig. 1).

**Table 1:** Demographics, history, and clinical features at randomization in 88 children with GAS pharyngitis

	Placebo, *N* (%)	Amoxicillin, *N* (%)
Patients	46 (52)	42 (48)
Age, mean (SD), year	7.0 (± 2.7)	7.8 (± 2.9)
≤ 6.0 years	16 (35)	15 (36)
6.1–10.0 years	22 (47)	17 (40)
≥ 10.1 years	8 (17)	10 (24)
Male	27 (59)	17 (40)
Origin		
Switzerland	14 (30)	15 (36)
Europe (except Switzerland)	24 (52)	23 (55)
Africa	6 (13)	4 (10)
Americas	1 (2)	0
Oceania	1 (2)	0
> 3 episodes/year of GAS pharyngitis	8 (17)	5 (12)
Identified source of contagion	13 (28)	6 (15)
Fever onset (SD), h	26.0 (± 10.8)	26.4 (± 11.8)
Presenting symptoms		
Sore throat	37 (80)	34 (82)
Tonsillar exudate	31 (67)	31 (74)
Tender/swollen anterior cervical lymph nodes	26 (58)	26 (62)
Anorexia	34 (74)	31 (74)
Irritability	29 (63)	27 (66)
GAS confirmed by culture	28/33 (85)	25/31 (81)

*GAS*, group A streptococcus

Continuous data are presented as mean (± standard deviation), and categorical data as frequency (percentage)

Throat swab culture results were only available for 33 patients in the placebo group and 31 in the amoxicillin group, with 28 (85%) and 25 (81%) participants confirmed as culture-positive GAS positive (Table 1).

**Fig. 2:**
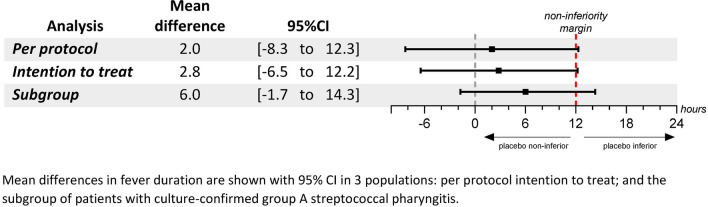
Mean difference in fever duration between amoxicillin and placebo groups

In the ITT population, the mean duration of fever was 21.7 h (SD = 21.8) in the amoxicillin group and 24.6 h (SD = 22.4) in the placebo group, and the mean difference in fever duration was 2.8 h (95% CI, − 6.5 to 12.2). The result was similar in the PP analysis, with a mean difference in fever duration of 2.0 h (95% CI, − 8.3 to 12.3). In the subgroup analysis of culture-confirmed patients, the mean difference in fever duration was 6.61 h (95% CI, − 4.5 to 17.7) (Fig. 2).

**Fig. 3:**
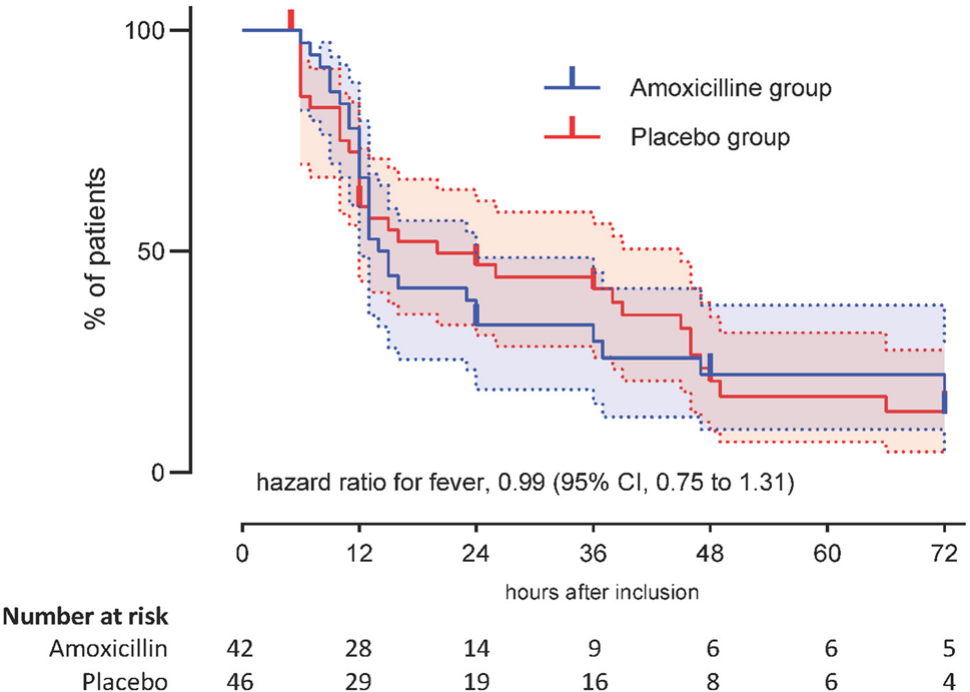
Kaplan–Meier survival curve illustrating estimates of the proportion of patients fever-free

**Fig. 4:**
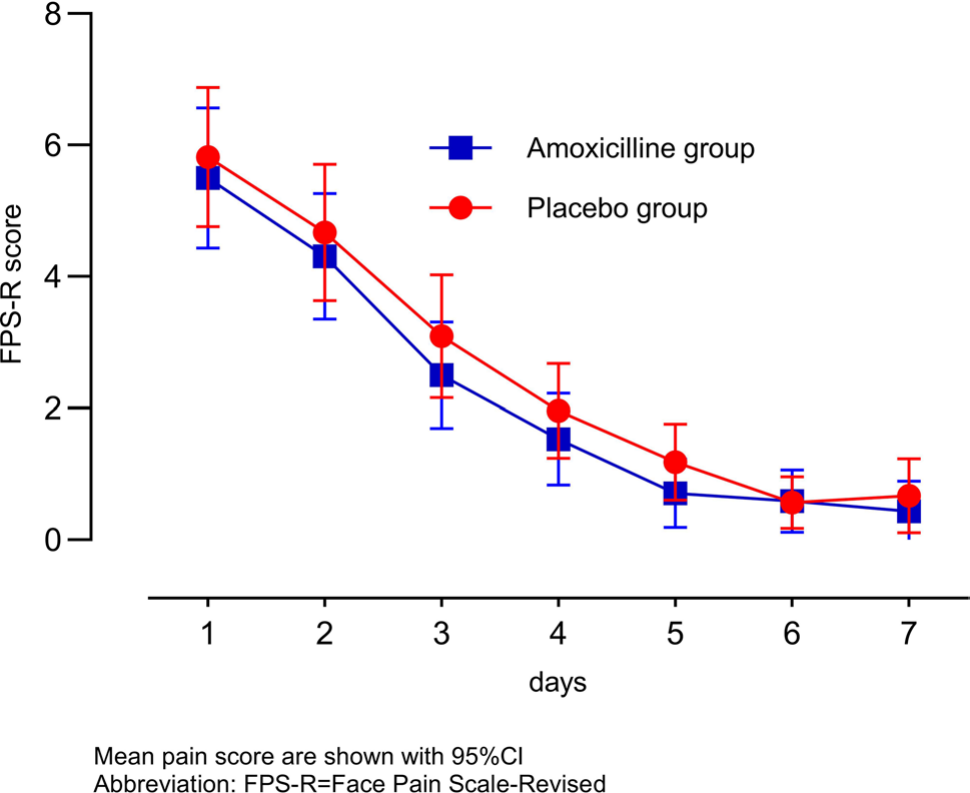
Daily throat pain score in the first 7 days after randomization to amoxicillin or placebo

As shown in the Kaplan–Meier curve (Fig. 3), all participants were afebrile or censored by day 3, and the groups’ curves overlapped (hazard ratio, 0.99; 95% CI, 0.75 to 1.31). The mean daily pain scores are presented by group in Fig. 4 and Supplementary Table 1.

The largest difference between the two groups was 0.5 points (95% CI, − 0.62 to 1.80) on day 3. No statistically significant difference was observed between the two groups with regard to the use of symptomatic treatment (paracetamol or NSAIDs) in 3 days following randomization (Table 2).

**Table 2:** Use of paracetamol or ibuprofen in the first 3 days following randomization

	Placebo (*n* = 34), *N* (%)	Amoxicillin (*n* = 31), *N* (%)	*P* value
Paracetamol			
Day 1	17 (50)	16 (51)	1.00
Day 2	17 (50)	13 (42)	0.62
Day 3	7 (21)	5 (16)	0.75
Ibuprofen			
Day 1	34 (100)	30 (97)	0.47
Day 2	33 (97)	29 (94)	0.60
Day 3	30 (88)	27 (87)	1.00

Treatment failures were observed in six participants (13%) in the placebo group (three scarlet fever, two acute otitis media, one retropharyngeal abscess) and two participants (5%) in the amoxicillin group (one scarlet fever, one acute otitis media) (*P* = 0.1). The relative risk of treatment failure was 2.15 (95% CI, 0.44 to 10.57) in the placebo group compared to the amoxicillin group.

In the PP analysis of the presence of symptoms 3 days after randomization, the two groups were comparable for most clinical manifestations (Table 3). The prevalence of asthenia was higher in the placebo group (*P* = 0.006). Logistic regression analysis that included clinical characteristics on day 1 did not identify any factors independently associated with this symptom.

**Table 3:** Clinical signs and symptoms at day 3 after randomization

	Placebo (*n* = 34), *N* (%)	Amoxicillin (*n* = 31), *N* (%)	*P* value
Throat pain			0.49
Absent	8 (12)	9 (14)	
Mild^a^	13 (20)	13 (20)	
Moderate^b^	10 (15)	9 (14)	
Severe^c^	3 (5)	0	
Asthenia	15 (44)	4 (12)	**0.006**
Headache	5 (15)	1 (3)	0.20
Abdominal pain	6 (18)	1 (3)	0.10
Anorexia	11 (33)	10 (32)	0.9
Conjunctivitis	2 (6)	0	0.49
Earache	2 (6)	1 (3)	1.00
Rhinorrhea	9 (27)	11 (35)	0.59
Cough	7 (21)	5 (16)	0.75
School absence	12 (37)	6 (21)	0.17

*P* values in bold indicate statistical significance

^a^Mild Faces Pain Scale-Revised (FPS-R) score ≥ 1 and < 4

^b^Moderate: FPS-R score ≥ 4 and < 8

^c^Severe: FPS-R score ≥ 8

During the 12-month follow-up period (Supplementary Table 2), no further suppurative or non-suppurative complications of GAS pharyngitis were reported in either group. A few episodes of streptococcal pharyngitis recurrence were observed and were equally distributed between the two groups.

In the subgroup analysis of culture-confirmed participants, we observed a higher prevalence of positive throat cultures at day 30 after randomization in the placebo group (12/18, 67%) compared with the amoxicillin group (2/20, 15%) (*P* = 0.002), with a relative risk of persistence of positive culture at 1 month of 4.44 (95% CI, 1.48 to 13.26).

## Discussion

In this randomized, double-blind, multicenter clinical trial including 88 children with GAS pharyngitis, antibiotic treatment was of limited benefit on the duration of fever and intensity of symptoms compared with placebo. However, it is commonly observed that when prescribing antibiotics for GAS pharyngitis, pediatricians are influenced by parental pressure and expectations for rapid symptom relief [29]. Our study findings show a marginal impact of amoxicillin on the duration of fever and intensity of pain and could help moderate such prescribing practices. Antibiotic overuse in children poses risks beyond antimicrobial resistance [30] and is also associated with adverse long-term health outcomes [31]. This calls for additional caution from physicians.

### Fever duration

Our findings showed that amoxicillin could reduce the duration of fever by a maximum of 12.3 h in the PP analysis and by a maximum of 12.2 h in the ITT analysis; these results are reassuringly consistent. Although they slightly exceeded the pre-established non-inferiority threshold of 12 h by 0.3 to 0.2 h (equivalent to 18 to 12 min), and therefore statistically, we must reject the hypothesis of non-inferiority of the placebo over amoxicillin, clinically, this excess may be considered inconsequential. Our result complements and enriches the existing literature, which only assessed the differences between antibiotic therapy and placebo after 3 days of treatment and not continuously, as in our study [32].

Interestingly, in the subgroup analysis of culture-confirmed GAS pharyngitis, the reduction in fever duration was greater (up to 17.7 h), thus suggesting a better efficacy of amoxicillin in culture-confirmed cases. However, this finding raises concerns about potential false positives among rapid antigen detection tests, which could dilute the effect of amoxicillin in cases of viral origin. The GAS rapid antigen detection tests may be falsely positive because of cross-reaction (e.g., with *Streptococcus milleri*) [33–35]. In addition, throat cultures may have been falsely negative because of non-viable GAS, resulting from suboptimal transport conditions of the swab specimen or growth inhibition by *Staphylococcus aureus* [34–37]. As the RADT was performed first, discomfort or opposition from the patient during the RADT might have led to less cooperation for the subsequent swab collection for culture, potentially compromising the quality of the sample and the detection of GAS by culture.

Nevertheless, this observation should be interpreted with caution because of the small sample size, which resulted in wider confidence intervals.

### Pain and other symptoms

Although our study was not powered for this outcome, we did not observe any difference in pain intensity between the two groups, and the 1.8-point maximum difference observed on day 3 may be clinically negligible. To our knowledge, this is the first study reporting on pain intensity in addition to its duration [38–41]. Of note, the knowledge gap on this aspect has already been raised in the literature [32]. The higher frequency of asthenia in the placebo group could be because amoxicillin attenuates the inflammatory response at an early stage, [42] which is probably responsible for this symptom. However, this alone may not be sufficient to justify antibiotic therapy, especially considering that patients who experience this symptom may be relieved with symptomatic treatment.

### Symptomatic treatment

Antibiotic treatment failed to reduce the use of paracetamol and NSAIDs during the first 3 days, consistent with a previous observation [41]. These data suggest that withholding antibiotics in managing streptococcal pharyngitis does not necessarily lead to a compensatory increase in symptomatic treatment.

### Complications

Withholding antibiotic treatment for GAS pharyngitis carries an increased risk of developing bacterial complications, such as otitis media and tonsillar abscess, in the following days. These findings are consistent with the results of the most recent Cochrane review [14] on the subject. However, it has already been suggested that these complications can generally be treated as soon as they occur [41]. Indeed, all patients in the placebo group who developed complications were promptly treated with antibiotics from the time of diagnosis and with a rapidly favorable outcome. In particular, the only case of tonsillar abscess occurring 3 days after randomization in a child in the placebo group was promptly treated with antibiotics and the patient recovered well without requiring surgery. Furthermore, the causal link between GAS pharyngitis and tonsillar abscesses has been questioned, and several studies have suggested that it is a complication of an infection of Weber’s glands rather than of pharyngitis [43, 44]. Therefore, an active follow-up with a stepwise approach to antibiotic prescription seems reasonable in countries where prompt access to healthcare and medication is not an issue.

No cases of rheumatic fever or post-streptococcal acute glomerulonephritis were observed during the 12-month follow-up period, although these two complications are now extremely rare in high-income countries (less than 1 per 100,000 per year [45–47]). The incidence of acute rheumatic fever has declined by 100- to 200-fold over the past decade in high-income countries, [48] possibly because of a decrease in rheumatogenic strains of GAS [49, 50]. In addition, withholding antibiotic treatment is not expected to influence the risk of post-streptococcal acute glomerulonephritis, as previous studies have failed to demonstrate a preventive effect of antibiotic treatment on this immunologically mediated complication [51].

### GAS eradication

Amoxicillin was more effective than placebo in eradicating GAS, as measured by throat culture 1 month after randomization. The three children in the amoxicillin group who were still positive 1 month after could possibly be GAS carriers [52]. In the placebo group, six participants had negative throat cultures 1 month after randomization, and GAS was likely eliminated by their immune system. Twelve participants were still culture-positive 1 month after randomization, and we were unable to confirm whether they subsequently became culture-negative. However, no complications were reported during the 1-year follow-up, and only one patient presented with a subsequent episode of GAS-positive pharyngitis 196 days after randomization. Because all patients who were still culture-positive 1 month after randomization were asymptomatic, we considered the persistence of GAS to have no clinical significance. However, this finding must be carefully analyzed, especially in the context of the recent resurgence of invasive GAS infections, and suggests the need for close epidemiological surveillance. Entry points for invasive GAS infections are uncertain, and there is ongoing discussion regarding the potential dissemination from the throat. While some authors [53, 54] have suggested that antibiotic treatment of streptococcal pharyngitis may reduce transmission and decrease invasive infections, more recent reports show that withholding antibiotic treatment for local GAS infections does not seem to be a risk factor for consecutive invasive GAS disease [55]. Furthermore, individuals with asymptomatic GAS infections have a low likelihood of transmitting the infection [56]. Therefore, along with other factors, Swiss health authorities have recently removed the mandatory school exclusion requirement until 24 h of antibiotic treatment for children with streptococcal pharyngitis, leaving the decision of school readmission to the discretion of the physician, based on clinical conditions.

### Limitations, strengths, and generalizability

The main limitation of this study is the sample size. However, a larger sample size would have likely resulted in a smaller (or at most equal) but not a larger 95% CI for the primary outcome. As such, we are inclined to cautiously consider our results as conclusive, as the 95% CI of our finding was nearly within the pre-defined non-inferiority margin of 12 h. This trial focused on a condition most often treated by primary care physicians. However, all three participating emergency departments also serve as primary care centers and see the same population as primary care physicians. In addition, the diagnostic approach in the two settings is similar and was reproduced in this study, which makes the results generalizable to all settings in countries with good access to medical care.

In contrast to previous studies on the treatment of GAS pharyngitis that enrolled patients based on the prevailing practice at that time, which was either clinical diagnosis without microbiological confirmation or positive culture of throat swab, our study is the first to use the rapid antigen detection test for GAS as an inclusion criterion. This approach enhances the generalizability and applicability of our findings to current practice, with the risk of false positive tests. Additional limitations include not performing emm typing, relying on parent reports for most outcomes, and the relatively high dropout rate, all secondary to parental will to initiate antibiotic treatment.

## Conclusions

In conclusion, our data suggest that the impact of antibiotic intervention on both fever duration and pain intensity may be marginal. Overall, our findings support a more restrictive attitude toward prescribing antibiotics. Effective patient monitoring and symptomatic treatment may be sufficient to manage GAS pharyngitis in children in high-income countries. Although this could imply an increase in medical consultations, the modest benefits of amoxicillin could be offset by concerns related to antibiotic overuse and the demonstrated effectiveness of symptom-based treatments. However, this approach, especially in light of the changing epidemiology of GAS infections post-pandemic, [57] warrants careful epidemiological surveillance.

## Supplementary Information

Below is the link to the electronic supplementary material.

Supplementary file1 (DOCX 20 KB)

## Data Availability

Deidentified individual participant data (including data dictionaries) will be made available in addition to study protocols, the statistical analysis plan, and the informed consent form. The data will be made available upon publication to researchers who provide a methodologically sound proposal for use in achieving the goals of the approved proposal. Proposals should be submitted to Professor Klara Posfay-Barbe, Klara.PosfayBarbe@hug.ch.

## Abbreviations

GAS: Group A streptococcus
PP: Per-protocol
ITT: Intention-to-treat
NNT: Number needed to treat
FPS-R: Faces pain scale-revised

## References

[1] Bisno AL, Gerber MA, Gwaltney JM Jr, Kaplan EL, Schwartz RH (2002) Practice guidelines for the diagnosis and management of group A streptococcal pharyngitis. Infectious diseases society of america. Clin Infect Dis 35(2):113–25 12087516 10.1086/340949

[2] Bisno AL (2001) Acute pharyngitis. N Engl J Med 344(3):205–211 11172144 10.1056/NEJM200101183440308

[3] Bryant A, Stevens D (2015) Streptococcus pyogenes. In: Saunders E, editor. Mandell, Douglas, and Bennett’s Principles and Practice of Infectious Diseases. 8th ed. Philadelphia. p. 2285–99

[4] Zwart S, Sachs APE, Ruijs GJHM, Gubbels JW, Hoes AW, de Melker RA (2000) Penicillin for acute sore throat: randomised double blind trial of seven days versus three days treatment or placebo in adults. BMJ 320(7228):150–154 10634735 10.1136/bmj.320.7228.150 PMC27262

[5] Denny FW, Wannamaker LW, Brink WR, Rammelkamp CH Jr, Custer EA (1950) Prevention of rheumatic fever; treatment of the preceding streptococcic infection. J Am Med Assoc 143(2):151–153 15415234 10.1001/jama.1950.02910370001001

[6] Robertson KA, Volmink JA, Mayosi BM (2005) Antibiotics for the primary prevention of acute rheumatic fever: a meta-analysis. BMC Cardiovasc Disord 5(1):11 15927077 10.1186/1471-2261-5-11 PMC1164408

[7] Shulman ST, Bisno AL, Clegg HW, Gerber MA, Kaplan EL, Lee G et al (2012) Clinical practice guideline for the diagnosis and management of group A streptococcal pharyngitis: 2012 Update by the Infectious Diseases Society of America. Clin Infect Dis 55(10):e86–e102 22965026 10.1093/cid/cis629 PMC7108032

[8] Van Brusselen D, Vlieghe E, Schelstraete P, De Meulder F, Vandeputte C, Garmyn K et al (2014) Streptococcal pharyngitis in children: to treat or not to treat? Eur J Pediatr 173(10):1275–1283 25113742 10.1007/s00431-014-2395-2

[9] National Institute for Health and Care Excellence (2018) Sore throat (acute): antimicrobial prescribing. NICE guideline [NG84]. https://www.nice.org.uk/guidance/ng84., accessed 10.11.2022

[10] Scottish Intercollegiate Guidelines Network (SIGN). SIGN Guideline Management of sore throat and indications for tonsillectomy. A national clinical guideline 2010. https://www.sign.ac.uk/media/1055/sign117.pdf, accessed 11.10. 2022

[11] Nederlands Huisartsen Genootschap. NHG-Standaard Acute keelpijn (derde herziening) 2015. https://richtlijnen.nhg.org/standaarden/acute-keelpijn#volledige-tekst, accessed 10.11.2022

[12] Belgian Antibiotic Policy Coordination Commission. Guide belge de traitement anti-infectieux en pratique ambulatoire 2021. https://www.health.belgium.be/sites/default/files/uploads/fields/fpshealth_theme_file/guide_belge_bapcoc_fr_2021_a4.pdf, accessed 11.10.2022

[13] Dixit J, Brar S, Prinja S (2022) Burden of group A streptococcal pharyngitis, rheumatic fever, and rheumatic heart disease in India: a systematic review and meta-analysis. Indian J Pediatr 89(7):642–650 34379301 10.1007/s12098-021-03845-y

[14] Spinks A, Glasziou PP, Del Mar CB (2013) Antibiotics for sore throat. Cochrane Database Syst Rev (11). 10.1002/14651858.CD000023.pub4 10.1002/14651858.CD000023.pub4 PMC6457983 24190439

[15] Swiss Society for Infectious Diseases. Pharyngitis - Guidelines 2023. https://ssi.guidelines.ch/guideline/2408, accessed 02.02.2024

[16] Gualtieri R, Barbe KP, Wagner N (2023) Streptokokkenangina im Kindesalter: Behandeln oder nicht behandeln? Paediatrica 500(1):4

[17] McIsaac WJ, White D, Tannenbaum D, Low DE (1998) A clinical score to reduce unnecessary antibiotic use in patients with sore throat. CMAJ 158(1):75–83 9475915 PMC1228750

[18] McIsaac WJ, Kellner JD, Aufricht P, Vanjaka A, Low DE (2004) Empirical validation of guidelines for the management of pharyngitis in children and adults. JAMA 291(13):1587–1595 15069046 10.1001/jama.291.13.1587

[19] Willis BH, Coomar D, Baragilly M (2020) Comparison of Centor and McIsaac scores in primary care: a meta-analysis over multiple thresholds. Br J Gen Pract 70(693):e245–e254 32152041 10.3399/bjgp20X708833 PMC7065683

[20] 2022 Nelson’s Pediatric Antimicrobial Therapy. Bradley JS, Nelson JD, editors: American Academy of Pediatrics; 01 Mar 2022

[21] Verolet C, Posfay-Barbe KM (2016) Revue Médicale Suisse : antibiotiques pour traiter la pharyngite à streptocoque chez les enfants en Suisse : est-ce encore utile ? Rev Med Suisse 12(506):334–337 27039456

[22] Hicks CL, von Baeyer CL, Spafford PA, van Korlaar I, Goodenough B (2001) The Faces Pain Scale-Revised: toward a common metric in pediatric pain measurement. Pain 93(2):173–183 11427329 10.1016/S0304-3959(01)00314-1

[23] Leelarasamee A, Leowattana W, Tobunluepop P, Chub-upakarn S, Artavetakun W, Jarupoonphol V et al (2000) Amoxicillin for fever and sore throat due to non-exudative pharyngotonsillitis: beneficial or harmful? Int J Infect Dis 4(2):70–74 10737842 10.1016/s1201-9712(00)90097-3

[24] Piaggio G, Elbourne DR, Altman DG, Pocock SJ, Evans SJW, CONSORT Group ft (2006) Reporting of noninferiority and equivalence randomized trials an extension of the CONSORT statement. JAMA. 295(10):1152–60 16522836 10.1001/jama.295.10.1152

[25] Jones B, Jarvis P, Lewis JA, Ebbutt AF (1996) Trials to assess equivalence: the importance of rigorous methods. BMJ 313(7048):36–39 8664772 10.1136/bmj.313.7048.36 PMC2351444

[26] European Agency for the Evaluation of Medicinal Products. Switching between superiority and non-inferiority - Scientific guideline London2000. https://www.ema.europa.eu/en/switching-between-superiority-non-inferiority-scientific-guideline#current-effective-version-section, accessed 03.08.2023

[27] Food and Drug Administration (FDA). Non-inferiority clinical trials to establish effectiveness guidance for industry 2016. https://www.fda.gov/media/78504/download, accessed 02.20.2024

[28] Boutis K, Willan AR (2011) Intention-to-treat and per-protocol analysis. Can Med Assoc J 183(6):696 10.1503/cmaj.111-2033 PMC3071397 21464181

[29] Park SY, Gerber MA, Tanz RR, Hickner JM, Galliher JM, Chuang I et al (2006) Clinicians’ management of children and adolescents with acute pharyngitis. Pediatrics 117(6):1871–1878 16740825 10.1542/peds.2005-2323

[30] GLASS method for estimating attributable mortality of antimicrobial resistant bloodstream infections. World Health Organization, Geneva; 2020. Licence: CC BY-NC-SA 3.0 IGO

[31] Duong QA, Pittet LF, Curtis N, Zimmermann P (2022) Antibiotic exposure and adverse long-term health outcomes in children: a systematic review and meta-analysis. J Infect 85(3):213–300 35021114 10.1016/j.jinf.2022.01.005

[32] Spinks A, Glasziou PP, Del Mar CB (2013) Antibiotics for sore throat. Cochrane Database Syst Rev 2013(11):CD000023 24190439 10.1002/14651858.CD000023.pub4 PMC6457983

[33] Rubin LG, Kahn RA, Vellozzi EM, Isenberg HD (1996) False positive detection of group A streptococcus antigen resulting from cross-reacting Streptococcus intermedius (Streptococcus milleri group). Pediatr Infect Dis J 15(8):715–717 8858682 10.1097/00006454-199608000-00021

[34] Johnson DR, Kaplan EL (2001) False-positive rapid antigen detection test results: reduced specificity in the absence of group A streptococci in the upper respiratory tract. J Infect Dis 183(7):1135–1137 11237843 10.1086/319286

[35] Cohen JF, Cohen R, Bidet P, Levy C, Deberdt P, d’Humières C et al (2013) Rapid-antigen detection tests for group a streptococcal pharyngitis: revisiting false-positive results using polymerase chain reaction testing. J Pediatr 162(6):1282–4,4.e1 23465407 10.1016/j.jpeds.2013.01.050

[36] Rubin LG, Mirkin GS (2000) Apparent false positive detection of group a Streptococcus antigen resulting from pharyngeal infection with a nonhemolytic Streptococcus pyogenes. Pediatr Infect Dis J 19(7):672–674 10917236 10.1097/00006454-200007000-00026

[37] Schroeder S, Procop GW (2000) False positive strep A antigen test. Pediatr Infect Dis J 19(11):1114–1115 11099106 10.1097/00006454-200011000-00028

[38] el-daher NT, Hijazi SS, Rawashdeh NM, al-Khalil IA, Abu-Ektaish FM, Abdel-Latif DI (1991) Immediate vs. delayed treatment of group A beta-hemolytic streptococcal pharyngitis with penicillin V. Pediatr Infect Dis J 10(2):126–30 1905799 10.1097/00006454-199102000-00010

[39] Nelson JD (1984) The effect of penicillin therapy on the symptoms and signs of streptococcal pharyngitis. Pediatr Infect Dis 3(1):10–13 6366769 10.1097/00006454-198401000-00004

[40] Taylor B, Abbott GD, Kerr MM, Fergusson DM (1977) Amoxycillin and co-trimoxazole in presumed viral respiratory infections of childhood: placebo-controlled trial. Br Med J 2(6086):552–554 329949 10.1136/bmj.2.6086.552 PMC1631450

[41] Zwart S, Rovers MM, de Melker RA, Hoes AW (2003) Penicillin for acute sore throat in children: randomised, double blind trial. BMJ 327(7427):1324 14656841 10.1136/bmj.327.7427.1324 PMC286321

[42] Kaplan EL, Top FH Jr, Dudding BA, Wannamaker LW (1971) Diagnosis of streptococcal pharyngitis: differentiation of active infection from the carrier state in the symptomatic child. J Infect Dis 123(5):490–501 5115179 10.1093/infdis/123.5.490

[43] Klug TE, Rusan M, Fuursted K, Ovesen T (2016) Peritonsillar Abscess: complication of acute tonsillitis or Weber’s glands infection? Otolaryngol Head Neck Surg 155(2):199–207 27026737 10.1177/0194599816639551

[44] Kordeluk S, Novack L, Puterman M, Kraus M, Joshua BZ (2011) Relation between peritonsillar infection and acute tonsillitis: myth or reality? Otolaryngol Head Neck Surg 145(6):940–945 21810775 10.1177/0194599811415802

[45] Coppo R, Gianoglio B, Porcellini MG, Maringhini S (1998) Frequency of renal diseases and clinical indications for renal biopsy in children (report of the Italian National Registry of Renal Biopsies in Children). Group of Renal Immunopathology of the Italian Society of Pediatric Nephrology and Group of Renal Immunopathology of the Italian Society of Nephrology. Nephrol Dial Transplant 13(2):293–7 9509437 10.1093/oxfordjournals.ndt.a027821

[46] Simon P, Ramée MP, Autuly V, Laruelle E, Charasse C, Cam G et al (1994) Epidemiology of primary glomerular diseases in a French region. Variations according to period and age. Kidney Int. 46(4):1192–8 7861716 10.1038/ki.1994.384

[47] Essop MR, Nkomo VT (2005) Rheumatic and nonrheumatic valvular heart disease. Circulation 112(23):3584–3591 16330700 10.1161/CIRCULATIONAHA.105.539775

[48] Olivier C (2000) Rheumatic fever—is it still a problem? J Antimicrob Chemother 45(suppl_1):13–21 10759358 10.1093/jac/45.suppl_1.13

[49] Shulman ST, Stollerman G, Beall B, Dale JB, Tanz RR (2006) Temporal changes in streptococcal M protein types and the near-disappearance of acute rheumatic fever in the United States. Clin Infect Dis 42(4):441–447 16421785 10.1086/499812

[50] Hersh AL, Jackson MA, Hicks LA (2013) Principles of judicious antibiotic prescribing for upper respiratory tract infections in pediatrics. Pediatrics 132(6):1146–1154 24249823 10.1542/peds.2013-3260

[51] Committee on Infectious Diseases American Academy of Pediatrics, Kimberlin DW, Barnett ED, Lynfield R, Sawyer MH (2021) Group A streptococcal infections. Red Book: 2021–2024 Report of the Committee on Infectious Diseases: American Academy of Pediatrics. p. 0

[52] DeMuri GP, Wald ER (2014) The group A streptococcal carrier state reviewed: still an enigma. J Pediatric Infect Dis Soc 3(4):336–342 26625454 10.1093/jpids/piu030

[53] Lean WL, Arnup S, Danchin M, Steer AC (2014) Rapid diagnostic tests for group A streptococcal pharyngitis: a meta-analysis. Pediatrics 134(4):771–781 25201792 10.1542/peds.2014-1094

[54] Tapiainen T, Launonen S, Renko M, Saxen H, Salo E, Korppi M et al (2016) Invasive group A streptococcal infections in children: a nationwide survey in Finland. Pediatr Infect Dis J 35(2):123–128 26440814 10.1097/INF.0000000000000945

[55] Erlacher R, Toepfner N, Dressen S, Berner R, Bösch A, Tenenbaum T et al. Are invasive group A streptococcal infections preventable by antibiotic therapy?: A collaborative retrospective study. Pediatr Infect Dis J 9900. 10.1097/INF.0000000000004403 10.1097/INF.0000000000004403 PMC11407772 38830130

[56] Martin J (2022) The Carrier State of Streptococcus pyogenes. 2022 Sep 5 [Updated 2022 Oct 4]. In: Ferretti JJ, Stevens DL, Fischetti VA, editors. Streptococcus pyogenes: basic biology to clinical manifestations [Internet]. 2nd edition. Oklahoma City (OK): University of Oklahoma Health Sciences Center. Chapter 18. Available from: https://www.ncbi.nlm.nih.gov/books/NBK587119/

[57] Massese M, La Sorda M, De Maio F, Gatto A, Rosato R, Pansini V et al (2024) Epidemiology of group A streptococcal infection: are we ready for a new scenario? Lancet Microbe 5(7):620–621 38663425 10.1016/S2666-5247(24)00071-5

