# Supplementary material for: Amoxicillin vs. placebo to reduce symptoms in children with group A streptococcal pharyngitis: a randomized, multicenter, double-blind, non-inferiority trial
Source: Eur J Pediatr. 2024 Aug 31;183(11):4773–82. doi: 10.1007/s00431-024-05705-1 (PMC11473506; doi:10.1007/s00431-024-05705-1)
Supplement: Supplementary file 1 — Supplementary file1 (DOCX 20 KB) [file 431_2024_5705_MOESM1_ESM.docx]

# Supplemental Tables

Gualtieri R, Verolet C, Mardegan C, Papis S, Loevy N, Asner S, Rohr M, Llor J, Heininger U, Lacroix-Ducardonnoy L, Pittet L, Posfay-Barbe K. Amoxicillin vs Placebo to Reduce Symptoms in Children with Group A Streptococcal Pharyngitis: A Randomized, Noninferiority Trial

**Supplemental Table 1.** Daily Mean Difference in Faces Pain Scale-Revised Scores for Throat Pain Between 2 Groups During the First Week After Randomization

**Supplemental Table 2**. Clinical Findings During Follow-Up Periods

# Supplemental Table 1

| **Day** | **mean difference** | **[95% CI]** |
| --- | --- | --- |
| **1** | 0.22 | -1.25-1.69 |
| **2** | 0.35 | -1.03-1.74 |
| **3** | 0.59 | -0.62-1.80 |
| **4** | 0.43 | -0.54-1.41 |
| **5** | 0.46 | -0.29-1.22 |
| **6** | -0.01 | -0.62-0.58 |
| **7** | 0.22 | -0.48-0.94 |

# Supplemental Table 2

|  | **Placebo (n=34)**  **N (%)** | **Amoxicillin (n=31)**  **N (%)** | ***P value*** |
| --- | --- | --- | --- |
| **7-15 days after randomization** |  |  |  |
| Recurrence of throat pain | 1 (3) | 1 (3) | *1.00* |
| Abdominal pain | 1 (3) | 1 (3) | *1.00* |
| Recurrence of fever | 0 | 1 (3) | *0.47* |
| Henoch–Schönlein purpura | 0 | 1 (3) | *0.47* |
| **16-31 days after randomization** |  |  |  |
| Viral pharyngitis | 0 | 1 (3) | *0.47* |
| Streptococcal pharyngitis with scarlet fever | 0 | 1 (3) | *0.47* |
| Headache | 1 (3) | 0 | *1.00* |
| **31 to 180 days after randomization** |  |  |  |
| Streptococcal pharyngitis | 1 (3) | 2 (6) | *0.60* |
| Viral pharyngitis | 1 (3) | 2 (6) | *0.60* |
| Cervical lymphadenopathy | 0 | 1 (3) | *0.47* |
| **180 to 365 days after randomization** |  |  |  |
| Streptococcal pharyngitis | 3 (9) | 4 (13) | *0.70* |
| Viral pharyngitis | 2 (6) | 2 (6) | *1.00* |
| Streptococcal pharyngitis with scarlet fever | 1 (6) | 0 | *1.00* |

Data are reported as frequency (percentage) at 7-15 days, 16-31 days, 31-180 days, and 180-365 days after randomization.
